# Supplementary material for: Cholinergic neuromodulation of inhibitory interneurons facilitates functional integration in whole-brain models
Source: PLoS Comput Biol. 2021 Feb 18;17(2):e1008737. doi: 10.1371/journal.pcbi.1008737 (PMC7924765; doi:10.1371/journal.pcbi.1008737)
Supplement: S1 Fig — A) Mean participation coefficient PCw (integration) and transitivity Tw (segregation). B-C) Transitions in the α and β axes. Dashed lines represent critical transitions. (PDF) [file pcbi.1008737.s001.pdf]

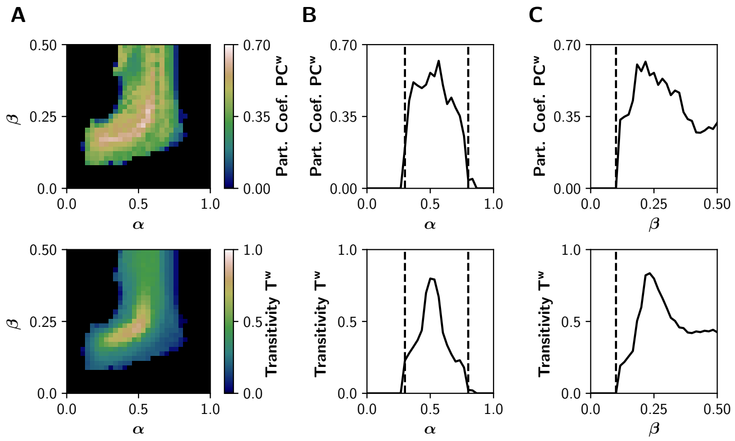


**S1 Fig. Alternative measures of network segregation and integration in the $(\alpha, \beta)$ parameter space.**

**A)** Mean participation coefficient $PC^w$ (integration) and transitivity $T^w$ (segregation). **B-C)** Transitions in the direction of $\alpha$ and $\beta$ axes. Dashed lines represent critical transitions.
