# Supplementary material for: Cholinergic neuromodulation of inhibitory interneurons facilitates functional integration in whole-brain models
Source: PLoS Comput Biol. 2021 Feb 18;17(2):e1008737. doi: 10.1371/journal.pcbi.1008737 (PMC7924765; doi:10.1371/journal.pcbi.1008737)
Supplement: S2 Fig — A) Global efficiency Ew (integration) and modularity Qw (segregation) of the graphs derived from the sFCs of the BOLD-like signals. B) Transitions in the α axis, for a fixed C1 = 0. C) Transitions in the C1 axis, for a fixed α = 0.5. Dashed lines represent critical transitions. (PDF) [file pcbi.1008737.s002.pdf]

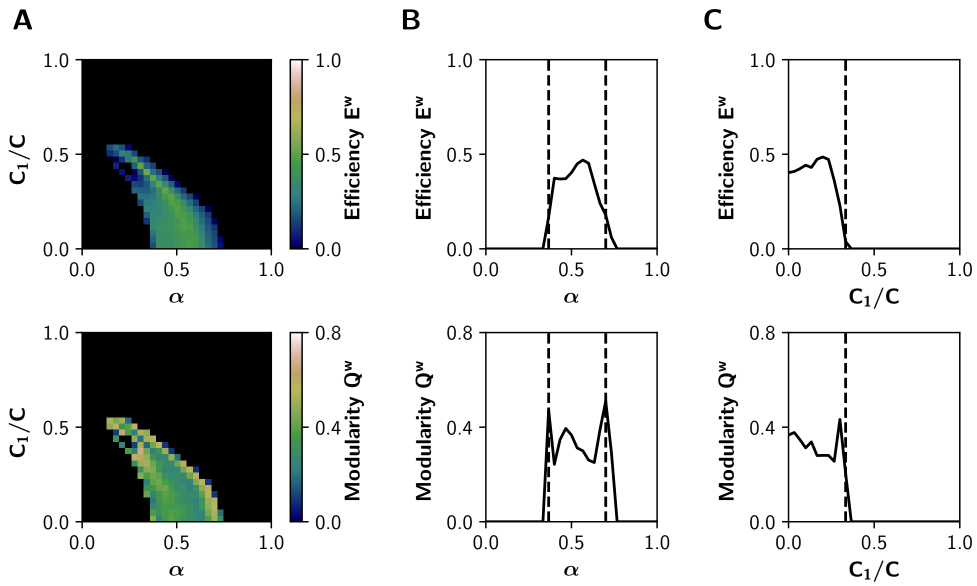

S2 Fig. Network features in the (α, C_1) parameter space.
A) Global efficiency E^w (integration) and modularity Q^w (segregation) of the graphs derived from the sFCs of the BOLD-like signals. **B)** Transitions in the direction of α axis, for a fixed $C_1 = 0$. **C)** Transitions in the direction of C_1 axis, for a fixed $\alpha = 0.5$. Dashed lines represent critical transitions.
