# Supplementary material for: Cholinergic neuromodulation of inhibitory interneurons facilitates functional integration in whole-brain models
Source: PLoS Comput Biol. 2021 Feb 18;17(2):e1008737. doi: 10.1371/journal.pcbi.1008737 (PMC7924765; doi:10.1371/journal.pcbi.1008737)
Supplement: S3 Fig — A-B) Mean participation coefficient PCw (integration) and transitivity Tw (segregation) with A) β = 0 and B) β = 0.4. C-D) Transitions in the α and r0 axes. Dashed lines represent critical transitions. (PDF) [file pcbi.1008737.s003.pdf]

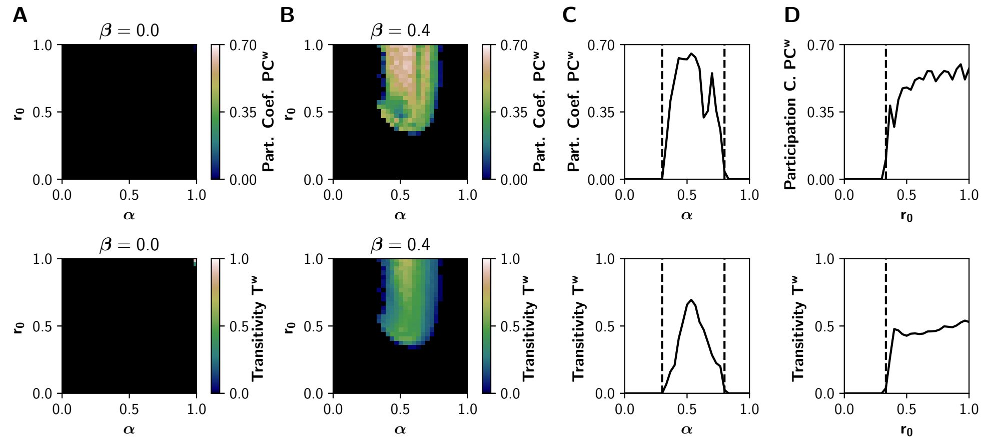

S3 Fig. Alternative measures of network segregation and integration in the (α, r_0) parameter space for $\beta = 0.4$.
A-B) Mean participation coefficient PC^w (integration) and transitivity T^w (segregation) with **A)** $\beta = 0$ and **B)** $\beta = 0.4$. **C-D)** Transitions in the direction of α and r_0 axes. Dashed lines represent critical transitions.
