# Supplementary material for: Cholinergic neuromodulation of inhibitory interneurons facilitates functional integration in whole-brain models
Source: PLoS Comput Biol. 2021 Feb 18;17(2):e1008737. doi: 10.1371/journal.pcbi.1008737 (PMC7924765; doi:10.1371/journal.pcbi.1008737)
Supplement: S4 Fig — A) Global efficiency Ew (integration) and modularity Qw (segregation) of the graphs derived from the sFCs of the BOLD-like signals. B) Transitions in the α axis, for a fixed r0 = 0.5 mV−1. C) Transitions in the r0 axis, for a fixed α = 0.5. Dashed lines represent critical transitions. Both coupling parameters change in parallel following the relationship β = 0.5α. (PDF) [file pcbi.1008737.s004.pdf]

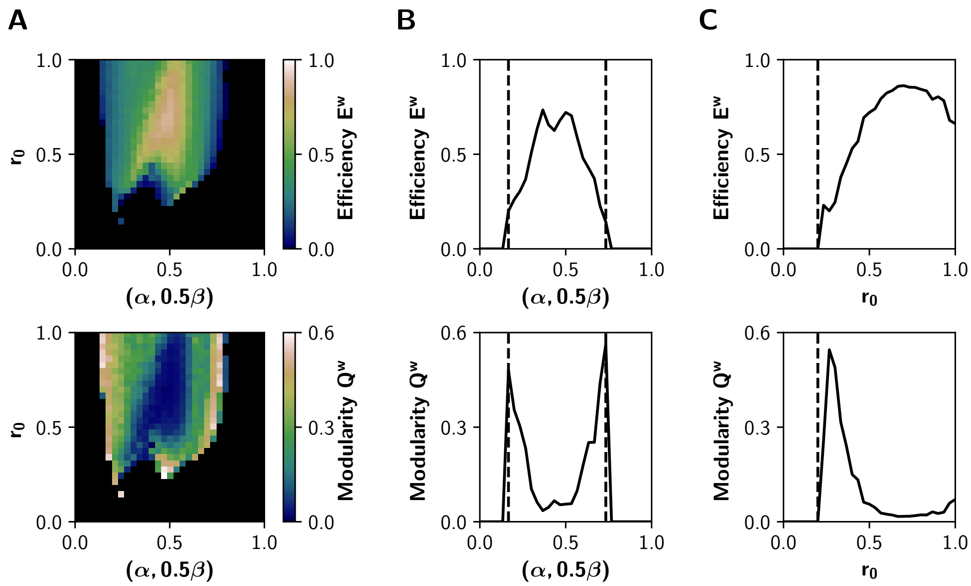

S4 Fig. Simultaneous effect of α, β and r_0 in network features.
A) Global efficiency E^w (integration) and modularity Q^w (segregation) of the graphs derived from the sFCs of the BOLD-like signals. **B)** Transitions in the direction of α axis, for a fixed $r_0 = 0.5$ mV^{-1}. **C)** Transitions in the direction of r_0 axis, for a fixed $\alpha = 0.5$. Dashed lines represent critical transitions. Both coupling parameters change in parallel following the relationship $\beta = 0.5\alpha$.
