# Supplementary material for: Cholinergic neuromodulation of inhibitory interneurons facilitates functional integration in whole-brain models
Source: PLoS Comput Biol. 2021 Feb 18;17(2):e1008737. doi: 10.1371/journal.pcbi.1008737 (PMC7924765; doi:10.1371/journal.pcbi.1008737)
Supplement: S5 Fig — A-B) Average phase synchrony R¯ measured over the EEG-like signals, and global efficiency Ew computed over the sFC matrices build using the BOLD-like signals. σ and μ reduces the increment of R¯ and Ew mediated by α and β. Both coupling parameters change in parallel following the relationship β = 0.5α. C-D) Phase synchrony R¯ and global efficiency Ew as a function of α and β, for different σ (left hand) and μ (right hand) values. (PDF) [file pcbi.1008737.s005.pdf]

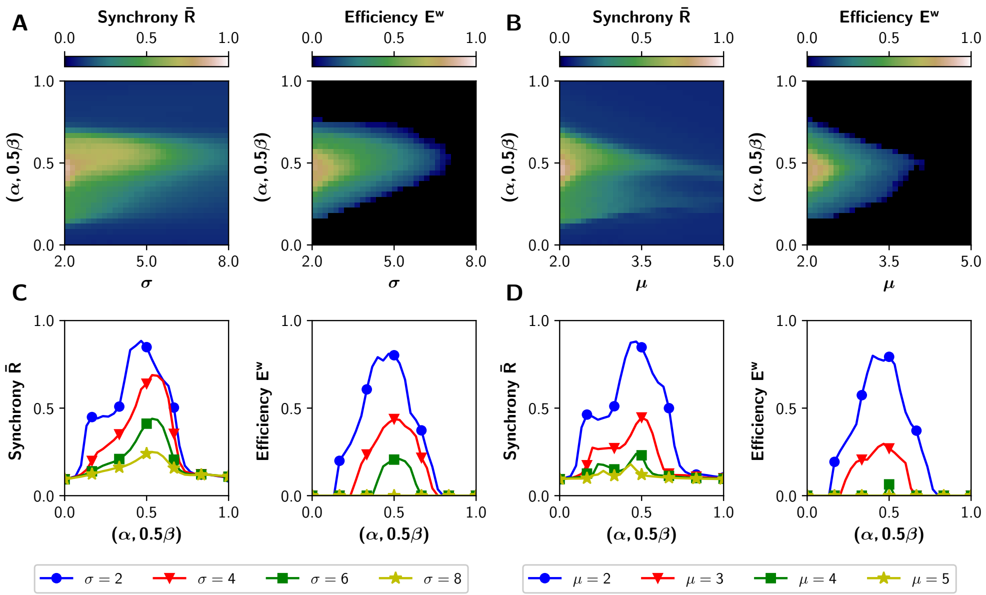

S5 Fig. Effect of the input standard deviation, σ, and mean, μ, in synchronization and integration.

A-B) Average phase synchrony $\bar{R}$ measured over the EEG-like signals, and global efficiency E^w computed over the sFC matrices build using the BOLD-like signals. σ and μ reduces the increment of $\bar{R}$ and E^w mediated by α and β. Both coupling parameters change in parallel following the relationship $\beta = 0.5\alpha$. **C-D)** Phase synchrony $\bar{R}$ and global efficiency E^w as a function of α and β, for different σ (left hand) and μ (right hand) values.
