# Supplementary material for: Cholinergic neuromodulation of inhibitory interneurons facilitates functional integration in whole-brain models
Source: PLoS Comput Biol. 2021 Feb 18;17(2):e1008737. doi: 10.1371/journal.pcbi.1008737 (PMC7924765; doi:10.1371/journal.pcbi.1008737)
Supplement: S6 Fig — A) Global efficiency Ew computed in the entire (α, β) parameter space. BOLD-like signals were computed using only the firing rates of pyramidal neurons. B) Ew calculated starting from a summation of the BOLD-like signals simulated using the firing rates of the three neural masses: pyramidal neurons, excitatory and inhibitory interneurons. C) Difference in the global efficiency ΔEw between the two matrices in the (α, β) parameter space. Green values correspond to near-zero difference between the matrices. There is not a noticeable difference between them. (PDF) [file pcbi.1008737.s006.pdf]

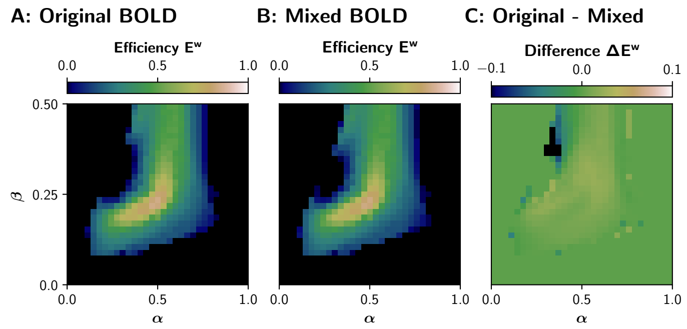

S6 Fig. Network integration computed from mixed BOLD-like signals.
A) Global efficiency E^w computed in the entire (α, β) parameter space. BOLD-like signals were computed using only the firing rates of pyramidal neurons. **B)** E^w calculated starting from a summation of the BOLD-like signals simulated using the firing rates of the three neural masses: pyramidal neurons, excitatory and inhibitory interneurons. **C)** Difference in the global efficiency ΔE^w between the two matrices in the (α, β) parameter space. Green values correspond to near-zero difference between the matrices. There is not a noticeable difference between them.
