# Supplementary material for: Cholinergic neuromodulation of inhibitory interneurons facilitates functional integration in whole-brain models
Source: PLoS Comput Biol. 2021 Feb 18;17(2):e1008737. doi: 10.1371/journal.pcbi.1008737 (PMC7924765; doi:10.1371/journal.pcbi.1008737)
Supplement: S7 Fig — Bifurcations of a single-node model with respect to the mean external input parameter p, at three values of β. Note that β = 0 corresponds to the original Jansen & Rit model. The value depicted in the y-axis is the variable x0. Red and black lines denote stable and unstable fixed points, respectively. Green solid lines and blue dashed lines represent stable and unstable periodic attractors, respectively (denoting the maximum and minimum values of the oscillation). At the right, sample time courses (3 seconds) of the EEG-like signal (C2 x1(t) − C4 x2(t)) at three values of p, and their corresponding power spectra below. (PDF) [file pcbi.1008737.s007.pdf]

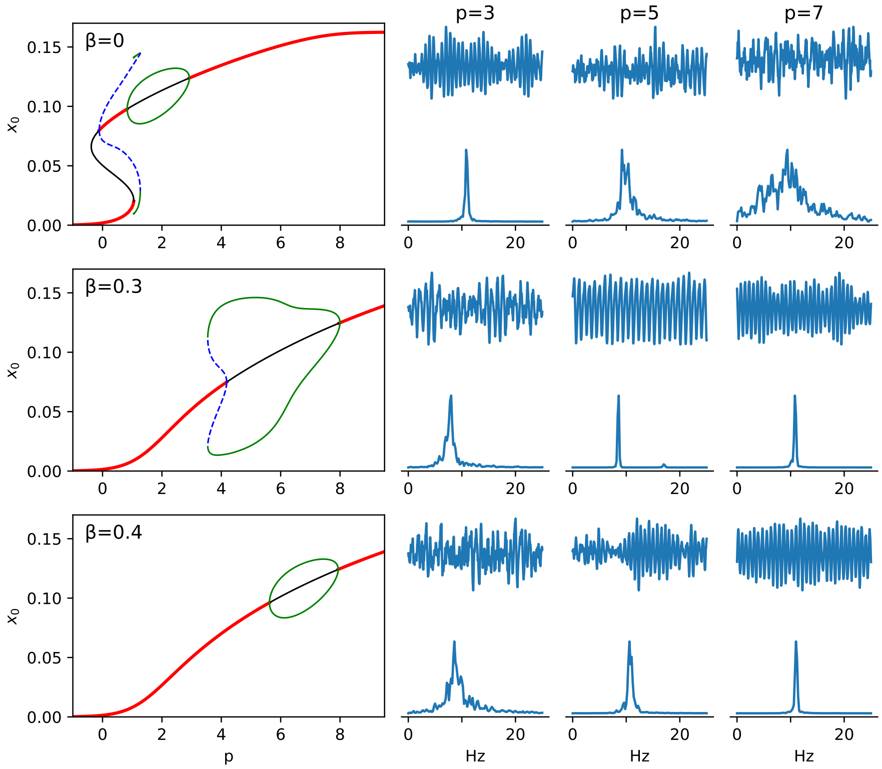

S7 Fig. Bifurcation analysis of the modified Jansen & Rit model and comparison to the original model.
Bifurcations of a single-node model with respect to the mean external input parameter p, at three values of β. Note that $\beta = 0$ corresponds to the original Jansen & Rit model. The value depicted in the y-axis is the variable x_0. Red and black lines denote stable and unstable fixed points, respectively. Green solid lines and blue dashed lines represent stable and unstable periodic attractors, respectively (denoting the maximum and minimum values of the oscillation). At the right, sample time courses (3 seconds) of the EEG-like signal $(C_2 x_1(t) - C_4 x_2(t))$ at three values of p, and their corresponding power spectra below.
