# Supplementary material for: Cholinergic neuromodulation of inhibitory interneurons facilitates functional integration in whole-brain models
Source: PLoS Comput Biol. 2021 Feb 18;17(2):e1008737. doi: 10.1371/journal.pcbi.1008737 (PMC7924765; doi:10.1371/journal.pcbi.1008737)
Supplement: S8 Fig — The frequency falls in the Theta range (4-8 Hz) of the EEG spectrum in both the A (α, β) and B (α, r0) parameter spaces. In the first case we fixed r0 = 0.56 mV−1, and in the second one β = 0.4. The frequency drop off matches with phase synchronization (see Fig 5). (PDF) [file pcbi.1008737.s008.pdf]

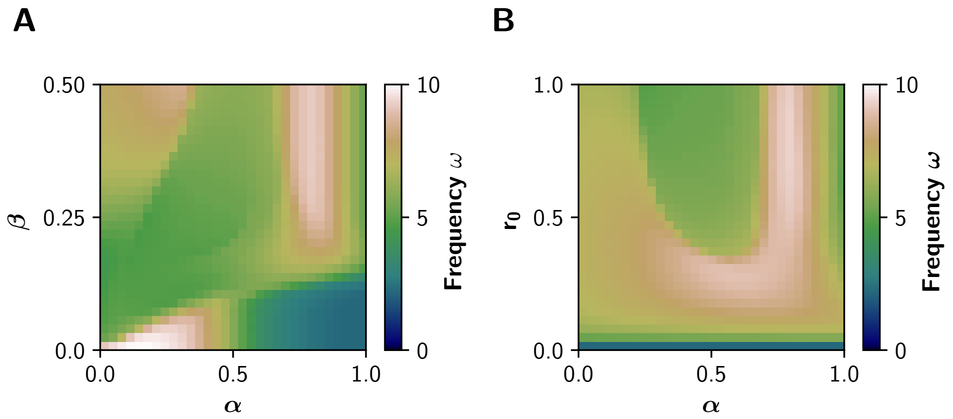

S8 Fig. Effect of neuromodulation on the mean oscillatory frequency ω. The frequency falls in the Theta range (4-8 Hz) of the EEG spectrum in both the **A** (α, β) and **B** (α, r_0) parameter spaces. In the first case we fixed $r_0 = 0.56$ mV^{-1}, and in the second one $\beta = 0.4$. The frequency drop off matches with phase synchronization (see Fig 5).
