# Supplementary material for: Cholinergic neuromodulation of inhibitory interneurons facilitates functional integration in whole-brain models
Source: PLoS Comput Biol. 2021 Feb 18;17(2):e1008737. doi: 10.1371/journal.pcbi.1008737 (PMC7924765; doi:10.1371/journal.pcbi.1008737)
Supplement: Attachment — Submitted filename: ReviewersResponse.pdf [file pcbi.1008737.s009.pdf]

Reply to Reviewers:
PCOMPBIOL-D-20-01747 - EMID:0c2242f72ea8e899
Cholinergic neuromodulation of inhibitory interneurons facilitates functional integration in whole-brain models

Carlos Coronel-Oliveros, Rodrigo Cofré, Patricio Orio

December 27, 2020

We would like to thank the referees for their critical reading and pertinent concerns, as they have allowed us to improve our manuscript. We have prepared a revised version of our manuscript, which includes all the reviewers' observations. Bold fonts are used for the referees' comments and regular fonts for our reply.

Reviewer 1

1) Reviewer #1: Thank you for inviting me to review this manuscript by Coronel-Oliveros and colleagues, in which the authors adapt a Jansen-Rit neuronal model to replicate and extend previous work relating the ascending neuromodulatory arousal system to network-level topological characteristics and temporal signatures of neural activity.

Can the authors please discuss what the 'excitatory interneuron' population represents in the Jansen-Rit model. The term 'interneuron' is typically used to describe locally-projecting GABAergic neurons (`http://doi.org/10.1146/annurev-neuro-070918-050421`). Although some of these neurons, by virtue of their inhibitory projections to other GABAergic neurons, are thought to disinhibit excitatory pyramidal neurons (e.g., `http://doi.org/10.1038/s41593-019-0508-y`), it is hard to know whether this is the population the authors are referring to. Alternatively, the excitatory interneuron population could reflect local (i.e., within column) recurrent activity, however this would imply a different set of properties (e.g., time-scales) and responsiveness (or not) to different classes of neuromodulatory neurotransmitters (see next point).

The reviewer raises a fair concern. Local excitatory interneurons correspond to nearby pyramidal cells in the cortical column - layer 5, the same layer as the principal pyramidal cells. See `https://doi.org/10.3389/fncom.2019.00054` for a reference, in particular Figure 2. According to the original model description found in subsection 2.1 of Jansen & Rit (1995): "The cortical column is modeled by a population of feedforward pyramidal cells, receiving inhibitory and excitatory feedback from local interneurons (i.e., other pyramidal, stellate or basket cells residing in the same column) and excitatory input from neighboring or more distant columns (Fig. 1)". We added, within the model description in the Results and Methods section, this brief explanation.

"excitatory interneuron population could reflect local (i.e., within column) recurrent activity". This in fact is represented, in our model, by the excitatory interneurons population: recurrent excitatory activity.

"however this would imply a different set of properties (e.g., time-scales) and responsiveness (or not) to (...) neurotransmitters". The reviewer was right, it is known that different kind of receptors are expressed in particular cell types within the cortical column (`https://doi.org/10.1093/cercor/bhr390` for an example related to nicotinic receptors). The intra-columnar inhibition and the responsivity to afferent inputs are mediated by both nicotinic –fast action– (`http://doi.org/10.1016/S0168-0102(00)00151-6`) and muscarinic (`http://doi.org/10.1126/science.281.5379.985`) receptors. In our model, we considered the effect of these receptors in the proposed mechanisms. For example, the increase of intra-columnar inhibition, modelled by the parameter β, reflects the increment of the inhibitory tone mediated by interneurons through nicotinic receptors (`http://doi.org/10.1016/S0168-0102(00)00151-6`). Regarding the timescale of

the neuromodulatory influence in increasing the intra-columnar inhibition, acetylcholine promotes this kind of inhibition in a faster timescale through nicotinic receptors expressed on inhibitory interneurons (http://doi.org/10.1016/j.neuron.2012.08.036).

Along these lines, I worry that the link between the cholinergic system and the inhibitory connection introduced in their model may not be as specific as the authors hope. Importantly, this depends on precisely how the authors (and others) conceptualize the excitatory interneuron population. If they are conceptualizing EI as VIP+ interneurons, then these cells have demonstrated responsivity to serotonin (http://doi.org/10.1523/JNEUROSCI.1869-10.2010), suggesting that the effects identified by the authors are not specific to acetylcholine. If the effects of EI are presumed to relate to recurrent pyramidal neurons, these connections could take the form of many different classes of cells (http://doi.org/10.1038/s41593-020-0685-8)

As we stated before, in our manuscript we consider that the excitatory interneurons population are local pyramidal neurons. The received disynaptic inhibition is mediated by inhibitory interneurons: see http://doi.org/10.1177/1073858412456743 for a reference of the disynaptic inhibition that we included in our model. We have clarified the nature of the excitatory interneurons within the model description in the Results and Methods sections.

Have the authors tested the stability/fit of their model following the addition of the new inhibitory connection? It's possible that, by adding this gain, the authors have fundamentally altered the fit to LFP data from the original study. This could potentially explain why the authors required different parameter combinations (e.g., $\beta = 0.4$ in Fig 4B) to recover the original results from Shine et al (2018).

Thanks for raising this issue. To characterize the behavior of the modified Jansen & Rit model, we performed a bifurcation analysis and observed typical time series at different values of β. As a bifurcation parameter we used $p(t)$, the external input. With $\beta = 0$, and confirming previous studies (Spiegler et al., 2010, http://doi.org/10.1016/j.neuroimage.2009.12.081) the model presents a couple of supercritical Hopf bifurcations between which a steady, 10Hz (alpha) oscillation develops. In addition, a mixture of subcritical Hopf, saddle-node and saddle bifurcations creates a small region where oscillations between 0.1 and 4.6 Hz appear. Introducing the β parameter causes these regimes to move to higher p values and around $\beta = 0.32$ the low-frequency component disappears and only the alpha-like oscillation prevails maintaining its frequency in the 10Hz range. More importantly, the bifurcations occur at higher p values, thus allowing the model to sustain oscillations when receiving a stronger external input now in the form of inter-columnar connections (our α parameter). In the new version of the manuscript, we include the bifurcation analysis as a Supplementary Figure.

Reviewer 2

Reviewer #2: The paper by Coronel-Oliveros et al. describes the effect of including local inhibitory circuits and their cholinergic modulation on segregation/integration balance in a modified Jansen-Rit model. These newly added local inhibitory coupling between interneurons is a variation of modelling inhibition-mediated control of the E/I balance which is an interesting approach to modelling neuromodulatory systems.

General comment: While the methods are profound, the graphs are of a high quality and the language of the manuscript is very understandable, the manuscript would benefit from clearly stating the essential message and sticking to its focus by restricting the number of exploratory analyses. In particular, while the introduction clearly states the interest in integration and segregation as a function of cholinergic action in inhibitory interneurons, also parameters such as Kuramoto order parameter, signal-to-noise-ratio, regularity, static FC, FCD, FCD speed, multistability (not even discussed in the methods), phase synchrony, mean oscillatory frequency and many more have been added. To me, the advantage of so many analyses and a wealth of companying very similar figures 2-5, 7 is not clear. Rather this approach seems like a rather exploratory analysis of all these parameters (that were taken from various computational neuroscience papers) without a clear focus and this approach not only dilutes the mean focus of the paper, makes all these values and their dependence on α and β much harder to interpret. The authors should restrict the parameters to those who answer the initial question of the manuscript and also rewrite the results and discussion section with a clearer focus to make their message more understandable. If more parameters than the original ones (segregation, integration) are chosen, it should be stated in the introduction on how they contribute to answering the original question.

We thank the reviewer for their suggestions. We hope the reviewer will find the new version of the manuscript more focused on the original question, the main results and the message we want to communicate. Following the suggestions of the referee, we reduced the number of measured variables in the Results section, making some figures simpler. We summarize the changes in the following list:

1) Figure 2: We now show only the Integration and Segregation measures.

2) Figure 4: Again we focus our results in Integration and Segregation.

3) We discuss the changes in the EEG-like signals in the third section of the Results. Now, we included the Synchrony and the Signal-To-Noise Ratio, but removed the Regularity Index from the manuscript, because it is redundant with the SNR.

4) In Figure 6, we shown only the results related to the BOLD-like signals: the Variance and Speed of the FCD.

5) We improved the motivation for measuring each metric different from segregation and integration.

With these changes, now the Results section is structured along three ideas: 1) The effect of the inhibitory cholinergic neuromodulation, in its interaction with the excitatory neuromodulation (Figs 2-3) and with the noradrenergic system (Fig 4), 2) the effect of neuromodulation in the fast (EEG) time scale (Fig 5), and 3) how the segregation/integration balance is related to dynamical variety of the network (Figs 6-7).

Detailed comments: The Results section includes many interpretations of the analyses (e.g. line 167, 161, 184, 172-183, etc). The authors should move these interpretations to the discussion section to make the Results section more concise.

As suggested, me moved all the possibles interpretations of the results to the Discussion section.

L. 162/203/etc: at times, different values when fixing α and β were chosen. What was the rationale between switching the fixed parameters? It would make sense to stick with the same fixed parameters across the different analyses and explain why these exact values were chosen.

Thanks for raising this issue. Because we are in a three dimensional space –considering the α, β and r_0 parameters–, we fixed the value of r_0 and β in different scenarios to facilitate the parameter swiping. In the (α, β) parameter space, we fixed $r_0 = 0.56$ because it constitutes the default value of the Jansen & Rit model; we also assumed that the cholinergic system modifies only the α and β parameters, and not r_0. In the (α, r_0)

parameters space, we selected $\beta = 0$ and $\beta = 0.4$ as two different values of inhibitory neuromodulation influence. The first ($\beta = 0$) corresponds the original Jansen & Rit configuration, while $\beta = 0.4$ was chosen to prevent the model to fall into highly synchronized regimes of activity. In fact, as they both obey to the cholinergic system, it is even more plausible that α and β changed together as we also explore in the Results (line 221 in the new version). Nevertheless, integration can also be observed with lower or higher of values of β (see for example the Supplementary Figure 4).

L. 498: what is the advantage to simulating a BOLD signal for these analyses? Why not stick with the EEG signal?

The reviewer has a good point, considering that the model election is a key step in computational modeling. We chose to work with BOLD signals, in parallel with EEG signals, because most of the experiments related to segregation and integration were originally conceived for fMRI-BOLD. See for example Cohen & D'Esposito (2016, `http://doi.org/10.1523/JNEUROSCI.2965-15.2016`), Shine et al. (2016, `http://doi.org/10.1016/j.neuron.2016.09.018`), and Wylei et al. (2012, `http://doi.org/10.1016/j.neuroimage.2012.06.079`). However, we chose the Jansen & Rit model that produces an EEG-like signal, because it allowed us to biophysically represent the influences of cholinergin and noradrenergic systems (through the input-to-output function). The model also incorporates mesoscopic properties (such as the excitation/inhibition balance). Therefore, we had to incorporate the extra step of converting the excitatory levels of activity to a BOLD-like signal as it has been done in other works (`http://doi.org/10.1016/j.cub.2018.07.083`).

Fig. 3: is along the lines of my first comment. I do not see the main message of this figure and how it contributes to answering the original question Fig 1 and 4 seem almost redundant to me- in my view, the manuscript would benefit from reducing the number of figures.

We agree with the reviewer in that we originally arranged the Results section and its figures in a way that made difficult to follow our central ideas. We hope that the overall changes we have made to the manuscript will fix this and will make easier to the reader to distill the main messages. Although we are keeping the same number of figures, several of them now have less panels and less information than in the first submission.
Regarding Figure 3, in our opinion it is important to show the BOLD-like signals and their corresponding Functional Connectivity matrices for different combinations of α and β as different regimes of integration and segregation emerge from them. Also, showing –at least once– an intermediate step in the analysis is a way to point out that our results are robust and not a singularity in the parameter space nor artifacts of the simulations. The same logic applies to Figure 6, where we have chosen to show to the reader the intermediate steps and some raw data for an analysis (FCD) that not everybody may be familiar with.
Figure 1 is the model description therefore it cannot be redundant with Fig. 4. If the reviewer was referring to Figures 2 and 4, they are not redundant because they represent different sections of the parameter space. While in Figure 2 the effect of the cholinergic system only is shown (with its roles in modulating excitation and inhibition), in Figure 4 we expand the analysis to include the noradrenergic system. As now we have restricted the first part of results only to show functional integration and segregation, we hope this will be easier to appreciate.

L. 209: with a wealth of parameters, the critical boundaries

Thanks for the suggestion, we changed to critical boundaries in plural. We are not sure whether your suggestion was also to remark that many parameters can cause critical transitions (and we fully agree with that), but we prefer to restrict our observation to what is shown in Fig. 2, not to confuse the reader.

Discussion: When reading the discussion, I found it hard to understand the main focus of it- partly it is reads like an enumeration of ideas that are only partly connected. As I said in my first comment, the manuscript would benefit from a more precise and restricted analysis and in a similar fashion from a more focussed discussion focussing on the original question.

Thanks for your constructive criticism regarding the Discussion. In the new version of the manuscript we have completely reformulated the Discussion section, focusing in the original question, the main results and the main ideas we want to communicate: 1) the dynamical consequences of the newly introduced inhibitory control loop, 2) a brief account of observations that further justify of our model, 3) comments on the relationship between the integration/segregation balance and behavioral performance; and 4) possible future avenues and some concluding remarks.

L. 298: Please sum up the main results in the first section of the discussion

As we mentioned before, we completely reformulated the Discussion section. In particular, following your suggestion we summarized our principal results in the first paragraph of this section.

L. 309: the referenced papers (11,19) do not include any analyses of integration. Which experiments do you mean exactly and what did they really show? So far, I have only seen this relationship in whole-brain models only. Please add experimental papers that were dealing with neuromodulation and segregation/integration.

The reviewer raises a fair concern. The inverted-U relationship between neurmodulation and functional integration was described, to the best of our knowledge, just in computational models, similarly to Shine et al. (`http://doi.org/10.7554/eLife.31130.001`). The link between neuromodulation and integration that we discussed is indirect: in-task performance follows an inverted-U function with neuromodulation (`http://doi.org/10.1146/annurev.neuro.28.061604.135709`, `http://doi.org/10.1016/j.pneurobio.2011.06.002`) and with functional integration (`http://doi.org/10.1162/netn_a_00042`). Our model suggests that this inverted-U function could be found between neuromodulation and functional integration. This constitutes an interesting point for future research. We clarified this issue in the Discussion section.

L. 332: which results back that up? I missed the inverted U across the large amount of figures. Here, I again reiterate to reduce the number of figures according to your main question and focus on the figures with the main message.

Thanks for the suggestion. We reduced the information of several figures in the new version of the manuscript. We back up the original results of Shine et al. (2018, Figure 2, `http://doi.org/10.7554/eLife.31130.001`), who reported an inverted-U relationship between the excitatory gain (given by the parameter gamma γ, in their case) and integration.

L. 402: here two more parameters are introduced that should be removed from the manuscript to sharpen the focus on the original question

As we removed several other network measures from the manuscript, we have chosen to maintain Functional Connectivity Dynamic analysis to show some dynamic consequences of segregation/integration. We realize that the increase in integration is linked with stable Functional Connectivity patterns over time. In the new version of the Introduction and Discussion, we motivate better the inclusion of the Functional Connectivity Dynamics Analysis.

Code/Data availability: The authors provided a github repo with the accompanying code to reproduce the simulations. While I did not run the simulations myself, I found the code to be very well written and understandable and well documented. In addition to the provided codes, it would be good to include the measures of integration and segregation into the code as these are the main variables of interest in the paper.

Thank you for the positive comments about our Github repository. We have improved the code and now is optimized using the Numba package. Additionally, we included code to compute the integration/segregation metrics used in this article. The code uses the Brain Connectivity Toolbox (Rubinov & Sporns, 2010, `http://github.com/fiuneuro/brainconn`) and Networkx (Aric et al., 2008, `http://networkx.org/documentation/stable//index.html`).

Reviewer 3

The authors Coronel-Oliveros, Cofré, and Orio, performed a parameter exploration of a whole-brain network model utilizing local dynamics from a neural mass model to describe the effects of neuromodulatory systems and functional segregation and integration in the brain on the source network level and the level of BOLD and EEG. The presented study heavily relies on Shine et al., 2019, and the modeling work Shine et al., 2018. The authors extended the modeling work of Shine et al., 2018 by using a different local dynamic model. The authors considered two neuromodulatory systems, the cholinergic and the noradrenergic system. Both are assumed to act uniformly in the brain at the level of cortical columns. The cholinergic system is also considered to modulate the connectivity via white-matter fiber tracts. Both systems are assumed to act independently and on a slower time scale than the local dynamics. In a first modeling study, the authors systematically varied the level of local connectivity of inhibitory to excitatory interneurons and the level of long-range connectivity and assessed integration and segregation by graph-theoretic measures. In a successive study, the authors performed a similar analysis. They varied the level of long-range connectivity and the variance of firing thresholds for fixed connectivity levels from inhibitory to excitatory interneurons. The main result is that the cholinergic system action on both the long-range connectivity and the inhibition of excitatory interneurons is needed to shift from an unsynchronized regime towards a coherent activity (integrated). The model predicts that the projection of inhibitory to excitatory interneurons is important for controlling the dynamics of a brain area. I appreciate that kind of modeling work. However, the paper in the present version misses a proper description of observed phenomena associated with neuromodulation through the cholinergic and the noradrenergic system. Effects are often too vaguely described, and it is not clear how they are reflected in brain signals such as EEG and BOLD. I also miss a convincing motivation for the used model. For instance, the term neuromodulation repeatedly appears in the text, but the associated parameters are constants and do not change with time in the model. For the reader, it is important to know how constant model parameters and neuromodulation go together. The authors show the effect of the model parameters on the graph-theoretic measures (efficiency and modularity) and dynamic functional connectivity to assess integration and segregation of functional network states. However, the authors do not show and mention any particular functional networks. I am curious to see the occurring networks and how meaningful they are. I unfortunately cannot recommend the manuscript in its current stage for publishing in Plos-CB.

We thank the reviewer for the positive comments about our manuscript. Following their suggestions, we modified the Whole-Brain model and justified better in the manuscript some key aspects of our modeling approach. We summarize the changes we made in the new version of the manuscript in the following points:

1) The content of information of the figures was reduced; the focus of Figures 2-4 relies now in the segregation/integration measures.

2) The Discussion section was completely rewritten more focused on the results and their interpretation.

3) We justify some models choices in the several parts of the Methods section.

In spite of resting-state networks being widely characterized in fMRI, our main objective was to study how neuromodulation manages the transition from segregated to integrated states. It could be very interesting and meaningful to characterize the functional networks in computational models but that was not our main objective. Indeed, it is possible that the transitions that we report could be observed using network topologies other than the empirical human connectome, and thus our main result may be independent from the specific functional networks... or not. This is something we plan to address soon.

Major comments: My reservations concern the model and the description of the neuromodulation systems, most of which can be addressed by improving and elaborating the text's description. The description of the cholinergic and the noradrenergic systems should be clearer and more consistent throughout the paper. On the one hand, the authors should elaborate more on the physiological aspect - why these systems are so important? On the other hand, the authors should better motivate neuromodulation's modeled action in the local dynamic model. From the author's description of the biophysical mechanisms, I could imagine other implementations for the action of both systems, for example, the cholinergic system : lines 56/57, increasing the excitability of pyramidal neurons" could be modeled by lowering firing threshold theta of pyra-

midal cells and by increasing PSP gain, that is, C_2, C_4, and C at pyramidal cells. Why have the authors decided to scale the input from other brain areas to describe pyramidal cells' increased excitability? In the model, input from other brain areas is linear in the PSP at pyramidal cells. I agree that "lines 70/71: pyramidal neurons become more responsive to stimulus from other distant regions respect to the stimulus of its own cortical column." However, is that equivalent to "increasing the excitability of pyramidal neurons"? Please elaborate.

The reviewer raises a fair concern. We improved the description of neuromodulatory systems in the Introduction. We also explain better our modeling choices in several passages of the text.

Regarding the effect of the cholinergic system over pyramidal neurons, the reviewer is right that increasing the global coupling, α, is not the same as increasing the excitability of pyramidal neurons. However, α does increase the responsiveness of pyramidal neurons to the rest of the network, thereby augmenting their activity. As an example, we wrote in Methods: "Although α does not control directly the excitability, increasing α amplifies the input to pyramidal neurons [18, 19]".

In addition, we would like to comment that several of our modeling decisions obey to making our work comparable to others'. There are lots of different modeling choices that we could have implemented in order to have (arguably) a better model, but then it would be difficult to attribute a new finding to a specific mechanism. The main novelty in our model is the inclusion of the homeostatic inhibitory mechanism and its neuromodulation; thus we tried to replicate everything else the way it has been published before –having as a special reference the work by Shine et al. (2018).

Lines 67/68, enhancing the activity and firing rates of dendritic-targeting GABAergic interneurons" should be modeled by $\beta * x_2(t)$ at excitatory interneurons as well as pyramidal cells. Why is the presented model $\beta * x_2(t)$ affecting excitatory interneurons only and not the inhibitory projections onto pyramidal cells?

We added a disynaptic connection described in Fino et al. (`http://doi.org/10.1177/1073858412456743`). In spite of sharing, in equation (5), the same inhibitory interneurons that target the principal pyramidal cells, we scaled the disynaptic connection by a parameter β that is independent of the projections from inhibitory interneurons to principal pyramidal neurons. We can make this assumption if we consider that the inhibitory interneurons, which provide the disynaptic inhibition, constitute a different population from the one that targets directly onto pyramidal neurons.

the noradrenergic system : "Increases responsiveness to input-driven activity respect to spontaneous activity and filters out noise." In my opinion, that action is better represented in the model by the scaling α of the connections between brain areas, also because the "lines 51/52: the effect is more pronounced between distant brain regions." How do the authors relate this noradrenergic effect to a slope change in converting postsynaptic potentials to the firing rate? A flat slope allows for a linear transfer of potential dynamics into rate dynamics. A steep transfer function restricts the dynamic range of the conversion. Therefore, a neural mass is more likely to be saturated: the saturated unexcited or in the saturated excited state. In both states, the neural mass is less sensitive. In general, the slope reflects the variance of the firing thresholds theta within a neural mass. That's why I am curious to know why the authors have decided to alter the slope of potential-to-rate function for all neural masses? Please explain.

The reviewer remarks an interesting point. We took inspiration from the effect of the neural gain in increasing the signal-to-noise ratio in single neurons (`http://doi.org/10.1126/science.2392679`). In our model, the increment of r_0 sharpens the discrimination between below- or above-threshold inputs, decreasing the response to the former (assumed to be unspecific noise) and increasing the response to the later. On the contrary, an increase of α causes an overall increase in the response to inter-regional inputs. As suggested by Servan-Schreiber et al. (`http://doi.org/10.1126/science.2392679`) and Aston-Jones & Cohen (`http://doi.org/10.1146/annurev.neuro.28.061604.135709`), the increment of the signal-to-noise ratio promoted by r_0 facilitates the synchronization of the neural masses in our whole-brain model, considering intermediates values of α, e.g., for $\alpha \in [0.3, 0.7]$. Also, this modeling choice was reinforced by the fact that the previous work by Shine et al. (2018) did it in the same manner.

The authors should elaborate on the model decision. Most of the cholinergic neurotransmission is known on the level of neurons but the authors used a neural mass model instead of a neuronal model. Neurotransmitters are not directly implemented in neural mass models. The associated neurotransmitters do act on different time scales (see, Shine, 2019). What are the neurotrans-

mitters that potentially drive the constant level changes in the presented model. The authors should answer the question of why the level of neural masses and large-scale brain networks is appropriate for studying neuromodulation and functional integration and segregation in general?

In fact, the model selection is not a trivial step. The rationale behind the model selection is the following:

1) Segregation and integration have been studied mainly, but not exclusively, at the macro-scale level, that is, in the Whole-brain. On the other hand, neuromodulators usually affect a large number of neurons inside a brain region, thus changing their mesoscale dynamics. The model we chose spans both the mesoscale (internal dynamics of a node representing a brain area) and the macro-scale in assembling the network with the human connectome.

2) To test our hypothesis about the neuromodulation of inhibitory circuits by acetylcholine, we needed to use a model that considers the interactions between different neural populations inside a mass. This made the Jansen & Rit model almost an obvious choice, because it explicitly models both the excitatory and inhibitory feedback loops usually found within cortical columns, that we needed to alter in order to represent cholinergic influences.

3) The complex cascades of cellular signaling, triggered by neuromodulators in a faster timescale, can produce, in a slower timescale, changes in the properties of the input-to-output function of neurons, as remarked in `http://doi.org/10.1016/j.neuron.2018.01.008`. In a similar way, we use the input-output function of neural masses to analyze the effect of neuromodulation in our Whole-brain model.

4) "What are the neurotransmitters that potentially drive the constant level changes in the presented model". We modeled, starting from the ideas of Shine et al. (`http://doi.org/10.7554/eLife.31130.001`, `http://doi.org/10.1038/s41593-018-0312-0`), the effect of acetylcholine and noradrenaline.

For instance, it is unclear how connectivity speed is derived from the human connectomes. Here, I guess, the authors confused transmission delay/time with transmission speed. Please clarify and describe how to derive values with unit 1/seconds from a distance (which distance measure was used?).

The reviewer is right, we confused transmission speed with transmission delays, additionally our model does not have time delays. We used a distance-dependent PSP time constant ('D' matrix, calculated as Euclidean distances between all brain areas) as a quick and possibly dirty way to represent transmission delays, arising from a constant transmission speed with different distances between brain areas. However, we understand the confusion this causes and later noticed that our results are mostly maintained with a uniform time constant for inter-area (long-range) synaptic connections. We do not refer, in the new version of the manuscript, to time delays.

It is also unclear why the long-range connectivity speed (delay) affects the characteristic time constant of (dendritic) excitatory postsynaptic potentials at pyramidal cells? The impulse response functions $h_E(t)$ and $h_I(t)$ are properties of local neural masses such as the pyramidal cells. In contrast, long-range connectivity is a network property. The characteristic time constant of postsynaptic potentials differs dependent on the target of synapses on the dendrite. Studies on the single pyramidal cells show that inhibitory interneurons target closer to the soma and excitatory interneurons more distal. Excitatory synapses from more distant areas target more distal and show a distinct characteristic time constant in the postsynaptic potentials. So I agree to the extent that long-range input is integrated with a different time, but I do not see the point of having a different time constant for different lengths of long-range connections. Please elaborate. Time delays tau would read $x_{3,ij}(t - \tau_{ij})$ in the equation system (1).

The reviewer is right. It is not biological plausible to have different characteristic time constants for different long-range EPSPs. As we mentioned before, we modified the model using only one characteristic time constant for the long-range EPSPs, and we repeated the simulations using this new value.

What are the model assumptions? The modulatory systems do show spatial organization (e.g., `http://doi.org/10.1073/pnas.1703601115`). Is that an approximation? I suggest adding paragraphs discussing model assumptions, expectations, data descriptions, predictions, and how to test these.

The reviewer raises a fair concern. It is known that both cholinergic and noradrenergic neuromodulatory

systems have a spatial organization (http://doi.org/10.1016/j.neuron.2014.07.002,http://doi.org/10.1073/pnas.1703601115). We are not including this spatial organization, but we remarked this issue in the Discussion. Furthermore, we clarified some of our model choices in several parts of the Methods section.

My specific comments (reading the manuscript from the beginning to the end). Line 3: What is an optimal behavioral outcome?

Thanks for noting this, it is not a minor issue. We should not have referred to “optimal”, we meant “coherent”. A coherent behavioral outcome is a behavioral response that is well-suited considering the current context. We changed the word “optimal” for “coherent”.

Line 5: This statement should be softened. There are also other potential candidates for describing state changes (multistability dynamics, structured flow on manifolds, etc.).

The reviewer is right remarking that other elements, such as multistability and manifolds, can sustain the wide repertory of brain states. However, both constitute substrates for network reconfiguration, but not mechanisms that allow the transitions from segregation to integration. Indeed, neuromodulatory systems can produce a switch between different brain states. Also, we did not modify the Author Summary, because we consider that this level of detail is not necessary for that section.

Line 8: The point that ”segregation/integration balance is impaired in several neuropsychiatric disorders” should be discussed in more detail in the main text. How is such an impairment of segregation/integration balance reflected in brain signals? What are the relevant disorders?

Although it is very interesting to discuss how the alterations in the segregation/integration balance are related to neuropsychiatric disorder, dwelling on this point may dilute the main message of our work. We added in the Discussion two references of segregation/integration impairments in Parkinson disease (http://doi.org/10.1007/s00415-015-7750-3) and in schizophrenia (http://doi.org/10.1038/npjschz.2016.14). There is also a general reference for whole-brain modeling to understand the segregation/integration imbalance in neuropsychiatric disorders (http://doi.org/10.1098/rsta.2016.0283).

Line 35: Please provide a reference for the “non-stationarity” of functional connectivity. Are fluctuations at rest non-stationary or non-linear? See, for example, dx.http://doi.org/10.33892Ffnins.2020.00493 [https://doi.org/10.3389/fnins.2020.00493].

Thanks for the suggestion. As remarked by Guan et al. (2020) resting-state activity is both non-linear and non-stationary. We included this reference in the Introduction, and also modified the related phrase.

Line 40: “Neuromodulatory systems provide a biophysical mechanism that enhances the dynamical flexibility.” What are these systems? Please provide examples? It appears to be a category of several systems that are capable of modulating neurons. Also, a definition of ”dynamical flexibility” is missing.

The term “dynamical flexibility” was not the most accurate. The idea is more general, and it is not exclusive of any neuromodulatory system. We changed the entire phrase to: “Neuromodulatory systems tune the firing properties of neurons, providing a mechanism to change the flow of information within the brain. Thus, neuromodulation constitutes a plausible mechanism that the brain employs to manage the transitions between different FC patterns.”.

Line 44:“Indeed, the cholinergic system increments” That is one specific neuromodulatory system. It reads like there is no other. Please summarise for the reader what the “cholinergic system” is and elaborate on the neurophysical role of this system and its elements.

We added a brief description of the cholinergic system in the Introduction: “The cholinergic system is involved in cognitive and attentional selectivity [21], and in the cerebral cortex the main source of acetylcholine are projections from the basal forebrain [23]. Acetylcholine increments the...”. Also, the physiological aspect of the cholinergic system was addressed in the Introduction.

“[Indeed, the cholinergic system increments...] That is one specific neuromodulatory system. It reads like there is no other”. The reviewer is right. We softened the claim and wrote the next phrase: “In that line, the cholinergic and noradrenergic systems have been proposed as candidates to influence the cognitive processing within the brain [20, 21], in spite of not being the unique neuromodulatory systems in the central nervous system that can tune the firing properties of neurons [19, 22].”

Line 49: What is the "noradrenergic system"? Please elaborate.

We added a brief explanation of the noradrenergic system in the Introduction: "On the other hand, the noradrenergic system is related to the exploratory behavior [20], and the principal source of noradrenergic projections to the cerebral cortex comes from the locus coeruleus [26]. Noradrenaline increases...".

Line 50: "input-driven activity" What is the input? Do you mean stimuli like visual stimuli? Do you mean any input that a neural population receives other than its intrinsic "spontaneous activity"? Please clarify.

We clarified this issue with a pair of short phrases in the Introduction: "the noradrenergic system increases the responsivity (or selectivity) of neuronal populations to input-driven activity (e.g., visual stimuli, inputs for distant brain areas relevant to tasks) with respect to spontaneous activity (or the internal state of the brain)."

Line 67: Please clarify the difference between"activity and firing rate"?

Thanks for pointing out this issue. The words activity and firing rates are redundant. We left only the term firing rates.

Lines 67 to 71: ".. and second, enhancing the activity and firing rates of dendritic-targeting GABAergic interneurons, an effect that promotes intra-columnar inhibition, reducing the local excitatory feedback to pyramidal neurons [23,26,27]." This reads like "reducing the local excitatory feedback to pyramidal neuron" is a necessary reaction of the increased "intra-columnar inhibition" That is not necessarily the case because the pyramidal cells are also affected by intra-columnar inhibition.

The reviewer is right, a "whole" intra-columnar inhibition would affect both the excitatory interneurons and pyramidal neurons. However, the effect of the disynaptic inhibition that we included in our model is more focused (`http://doi.org/10.1126/science.281.5379.985`). We modified the phrase to "firing rates of dendritic-targeting GABAergic interneurons, an effect that promotes a focused intra-columnar inhibition"

Lines 89 to 91: "... excitatory gain, which increases the inter-columnar coupling. This gain mechanism is mediated by the action of the cholinergic system in pyramidal neurons, principally but not exclusively, and increments pyramidal excitability [10, 11, 22]." Is it not the noradrenergic system that acts on a large-scale between brain areas, as mentioned before?

The reviewer is right. The phrase is not accurate enough. In our model, both the coupling and the filter gain contributes to increasing integration. In fact, we wrote this in the Introduction: "Therefore, a complex interaction between the cholinergic and noradrenergic system seems to manage the balance between integration and segregation. Using a whole-brain model, Shine et al.[31] showed that neuromodulation...". Also, we modified the phrase of lines 98-99 (previous manuscript) to: "The increase of the signal-to-noise ratio by the noradrenergic system, alongside with the increment of global coupling, can promote functional integration".

Lines 95 to 99: "Finally, we incorporated a "filter gain," that increments the pyramidal neurons sigmoid function slope [11]. The noradrenergic system mediates this last gain mechanism; it acts as a filter, decreasing (increasing) the responsivity to weak (strong) stimuli [15,17], boosting signal-to-noise ratio and promoting integration [10]." The described actions are local and equal for all neural masses. Still, the effect is described to be long-range "lines 51 to 52: This effect is more pronounced between distant brain regions, in which structural connectivity is relatively low, promoting functional integration." Please clarify.

This is an important point to clarify. The filter gain r_0 does not control the global coupling, as the parameter α does. However, increasing r_0 has an effect on the signal-to-noise ratio, as we remarked above. When neural masses are more sensitive to neural activity than noise, the synchronization likelihood of the system increases. We remark again that this modeling choice is not ours only, it has been used before both in the interpretation of the data and in the modeling of neuromodulatory influences.

Lines 120/121: The time delays are not defined in the Materials and Methods section. If time delays are defined by the distance between brain areas divided by a speed, please clarify and discuss the assumed speed (is it a spatially invariant constant). What distance measure was applied (Euclidean as a lower bound proxy or mean streamline length?). Please elaborate.

As our representation of transmission delays turned out to be confusing and was not needed for the reproduction

of our results, we do not refer anymore to the time delays.

Page 4, Fig. 1. "The cholinergic system has a multiplicative effect on the sigmoid function. α amplifies pyramidal neurons' response to other columns' input" What do the authors mean exactly with multiplicative effect on the sigmoid function? Please explain in the main text? Please clarify "response"? Do the authors refer to postsynaptic potentials or firing rate?

In the original framework proposed by Shine et al. (`http://doi.org/10.7554/eLife.31130.001`,`http://doi.org/10.1016/j.tics.2019.04.002`), controlling the response gain is equivalent to scaling the global coupling of the FitzHugh-Nagumo neural network. However, in our case modifying the coupling between pyramidal neurons is not equivalent to directly changing the properties of the sigmoid function of pyramidal neurons. Instead, increasing α has an indirect effect on pyramidal neurons excitability, in a similar way to the external input $p(t)$. The reviewer is right in remarking this issue. We clarified it in the new version of the manuscript.

Lines 127 to 139: The authors should emphasize the model parameters and that these are constant levels for each simulation. I understand the presented model in that way that there is no neuromodulation. The model parameters α, β, and r_0 are constants and are no functions of time as readers might expect from "neuromodulation." The authors should highlight time scales, separate them, and why and under which circumstances the systematic exploration of constant parameters matters. Because this is so important for the modeling work, this should be mentioned and discussed at several stages in the manuscript.

The reviewer raises an interesting point. We are simulating the effects of tonic neuromodulation, in the same spirit as Deco et al. (2018, `http://doi.org/10.1016/j.cub.2018.07.083`) and Shine et al. (2018, `http://doi.org/10.7554/eLife.31130.001`). We remarked this issue in the new version of the manuscript within the Discussion section: "Our model considers neuromodulation to be static, that is, the parameters α, β, and r_0 do not change over time, as in tonic neuromodulation. Among the possible improvements to our model, there is the addition of the release and reuptake dynamics of neuromodulators, as in Kringelbachet et al. [74] or the characterization of the dynamics under acute neuromodulation 'pulses'."

Lines 242 to 244: There is a difference between noise and chaos in the model. A noise process drives the model with predefined moments. Chaos can occur due to the model's complexity and the coupling in the network (see `http://doi.org/10.1371/journal.pcbi.1002298` and `http://doi.org/10.1016/j.neuroimage.2016.02.015`). Although the applied measure does not distinguish between noise and chaos, the system's ability to show deterministic chaos should be discussed. Whereas the noise process represents an additional dimension and something 'unknown' extrinsic, the deterministic chaos is intrinsic and produced and maintained by the system itself.

We agree with the reviewer in chaos and noise being completely different in origin as well as in the effects they may have on the system. Moreover, the Regularity measure does not discriminate between noise and chaos. To avoid confusion, we have removed the Regularity measure and any mention of chaos or noise related to it.

Lines 259 to 297: The authors have to define the term criticality? Is it a statistical term, or does it correspond to deterministic mechanisms such as bifurcations that occur in the local dynamic model? Please elaborate.

In our whole-brain model, we used the term critical transitions to describe points in the parameter space, where the network transits from unsynchronized to synchronized behavior. The 'critical parameters' α, β and r_0 constitute the bifurcations parameters that cause the system to transit between the unsynchronized and synchronized regimes of activity.

Line 299 to 306: The authors refer to experimental findings based on the action of nicotinic acetylcholine (20,23,27) and somatostatin receptors (26) on spiking single neurons. How do these electrophysiological findings translate into the hypothesis that "cholinergic neuromodulation of the inhibitory interneurons (that suppresses the local 300 excitatory feedback to pyramidal neurons) facilitates functional integration?" Moreover, how can the utilized mesoscopic - large-scale level of neural masses and long-range connectivity help test the hypothesis. Why do the authors use forward models for EEG and BOLD? Do EEG and BOLD data exist supporting the hypothesis linked to the electrophysiological findings?

Thanks for raising this issue. We take inspiration from the aforementioned references to define the mechanisms in our model related to neuromodulatory systems. At the macro-scale level, we commented about some

experimental works in which fMRI recordings were taken under the effects of cholinergic and noradrenergic agonists. In the manuscript, some of the references correspond to http://doi.org/10.1016/j.neuroimage.2012.06.079, http://doi.org/10.1016/j.neuron.2016.09.018, http://doi.org/10.1162/netn_a_00042. The main idea in simulating BOLD-like signals is to link our results to real experiments, which were originally conceived for fMRI. In spite of the Jansen & Rit model being a model of EEG, we chose it because it incorporates mesoscale properties that other models lack (see for example http://doi.org/10.1073/pnas.1905534116).

Line 307 to 310: The references 11 and 19 are reviews, so I wonder which extend the presented model can explain the, in refs 11 and 19, discussed experimental data. Does the model explain more than the already described and modeled inverted U-shape (10,18)?

The reviewer raises a fair concern. The inverted-U relationship between neuromodulation and functional integration was described, in our knowledge, just in computational models, similarly to Shine et al. (http://doi.org/10.7554/eLife.31130.001). The link between neuromodulation and integration that we discussed is indirect: in-task performance follows an inverted-U function with neuromodulation (http://doi.org/10.1146/annurev.neuro.28.061604.135709, http://doi.org/10.1016/j.pneurobio.2011.06.002) and with functional integration (http://doi.org/10.1162/netn_a_00042). Our model suggests that this inverted-U function can also be found between neuromodulation and functional integration. This constitutes an interesting point for future research. We clarified this issue in the Introduction and Discussion. For example, in the Discussion we wrote a paragraph which starts with the next phrase: "From an experimental point of view, the inverted-U relationship between neuromodulation and integration that we are reporting in our whole-brain model, has not been observed [...]".

"Does the model explain more than the already described and modeled inverted U-shape". We wanted to reproduce the inverted-U function. The difference in our approach relies on the inclusion of the inhibitory gain β. The parameter β not only follows an inverted-U relationship with functional integration but also the extent of integration mediated by α and r_0 depends on the inhibitory gain driven by β. To study the contribution of inhibitory interneurons alone is of relevance, considering that alterations in the excitation/inhibition balance may impair the segregation/integration features of the healthy brain (http://doi.org/10.1098/rsta.2016.0283).

Lines 317/18: Again, the modes as presented do not include time delays. In the model, the transmission times of long-range connections determine the local characteristic time scale of the excitatory postsynaptic potentials at the receiving pyramidal neurons. Here, the authors should give motivation for that implementation in the model. To me, it does not sound biophysically plausible.

The reviewer is right, our model does not include time-delays. We have removed the distance-dependent time constants from the model.

Lines 449: How important are the mean and variance of the noise process for the results? What is the effect of noise?

The parameter σ, which controls the standard deviation of $p(t)$, has an effect on the likelihood of synchronization. Our results did not change qualitatively with σ, but the magnitude of the synchronization/integration decreases with the parameter. In the same manner, the mean μ of the input $p(t)$ has an effect in decreasing the integration and synchronization. We extended this idea in the first paragraph of the Whole-Brain Neural Mass Model section in Methods. Also, we imclude one Supplementary Figure related to the effects of μ and σ.

Lines 465 to 468: Do the authors really mean speed here? The physical SI-unit for speed is meter/second. So I wonder, how is the speed (m/s) derived from the connectome? Usually, the distance is decided by a constant speed to approximate transmission delays. However, to include transmission times as characteristic time constants in the ODEs is also not correct as these are two different things. The characteristic times in the ODEs, in fact, $h_{E,I}(t)$ represent local properties of the postsynaptic responses to synaptic input and should not vary for different incoming connections. Please elaborate. These points need to be clarified. What is the interpretation of the distances between brain areas here? Are we talking about Euclidean distances or mean streamline lengths between brain regions?

The reviewer is right. It is not biophysically plausible to have different characteristics time constants for long-range EPSPs. In the new version of the manuscript we clarified this issue, and now we used an unique characteristic time constant for long-range EPSPs.

Pages 13 The equations in the Materials and Methods section should be consistently numbered. In the presented manuscript are 17 equations, but only three equations have numbers.

In the previous version of the article we followed the convention of numbering only the equations that are referenced in the text. Although PLoS Computational Biology does not specify a policy on this issue, we now have numbered all the equations.

Page 13, equation (1):There are four sets of 2nd-order ODEs. For better understanding, it is worth describing their function. I. The first two equations are for the excitatory projections of pyramidal cells onto both interneurons. II. The second pair of equations is for the excitatory projections of excitatory interneurons onto pyramidal cells. Here we see, that the external input is assumed to be excitatory and share the characteristic time constant of intra-areal excitatory projections at the pyramidal cells. III. The third pair of equations is for the inhibitory projections of inhibitory interneurons onto pyramidal cells. IV. The fourth pair is very similar to the I pair of equations only differs in the scales and is meant to explain the excitatory long-range projections of pyramidal cells in distant areas onto target pyramidal cells.

Thanks for the suggestions. We added a more detailed explanation of the Jansen and Rit equations, for improving the understanding of the model. Also, we changed the last pair of equations considering that we employed, in the new version of the manuscript, a unique characteristic time constant for long-range EPSPs.

Here the time constants depend on the length of incoming connections, for which an explanation is missing. Please elaborate.

To build the matrix D we employed the distance between the centroids of each region defined in the AAL parcellation. The entries of D are inversely proportional to the distances between brain regions. However, as we remarked above it is not biophysically plausible to include different characteristic time constants for the long-range EPSPs, so we chose a unique time constant for all the long-range connections.

The notation of the incoming activity to y_0 and y_3 are identical (within the sigmoids). This becomes circular with inserting $z_i(t)$ (the unnumbered equation below). I guess, the input to y_3 should read $(C_2 x_{1,j} - C_4 x_{2,j} + C\alpha z_j)$ because the sigmoid looks backward into the source. That's why the equation for the average input should also be adapted to, for instance, $z_{a,b} = sum_{b=1}^{n} M_{ab} x_{3,ab}$, where a,b are indices of brain areas and b is the source whereas a is the target index.

Thanks for the suggestion. First, we modified the equations and used a unique value for the inverse of the characteristic time constant, for long-range connections, instead of the matrix D as in the previous version of the manuscript. The equations y_0 and y_3 may look identical because they represent the output of pyramidal neurons, which is the same independently of the targets. The difference relies on the two different EPSPs blocks that we used to integrate the outputs of pyramidal neurons: one for the local connections to both interneurons, and another for the long-range connections with distant pyramidal neurons. As explained before, both occur with different characteristic time constants (a and $\overline{a}$, respectively).

The authors should elaborate on the fact that the connectivity weights are normalized individually per receiving brain area. Why is that? The equation for the average input can be simplified $M_{ab} = M_{ij}/sum_j M_{ij}$, where M_{ij} is the weight as presented in the manuscript. An overall normalization by a scalar uniformly applied to all entries, for instance, the maximum in-strength sounds more plausible. By using an input wise normalization, the relative weights between receiving brain areas are lost.

This is a very interesting question. The normalization that we employed is a local normalization based on the strength of individual nodes. The local normalization constitutes a mechanism of homeostatic plasticity which equalizes the excitatory inputs that the nodes receive while preserving the structural topology. This is a very common assumption in network models (see `http://doi.org/10.1371/journal.pcbi.1004007` as an example) and it has been reported that this mechanism improves the fit of a whole-brain mesoscopic model to empirical fMRI data, leading to a better estimation of the Functional Connectivity (`http://doi.org/10.1038/s41598-018-33923-9`). In the new version of the manuscript, we change the notation of the structural connectivity matrix from M to $\widetilde{M}$, the later being the normalized version of M. In the Methods section, we explain the rationale of the normalization procedure of the structural connectivity matrix.

The equations show that the inhibitory interneurons have two targets: the pyramidal cells and the excitatory interneurons. The authors motivated the scaling of inhibitory activity as neuro-modulation. Why is it then that only the inhibitory postsynaptic potential at the excitatory

interneurons are scales but not the inhibitory postsynaptic potential at the pyramidal cells? However, the source of activity is identical? This modeling choice must be better motivated.

As we clarified before, we can make this assumption if we consider that the inhibitory interneurons, which provide the disynaptic inhibition, constitute a different population from the one that targets directly onto pyramidal neurons.

Line 473: the maximum firing rate zeta should be in 1/s it's a rate, not a frequency.

The thank the reviewer for pointing out this issue. We have changed the units as suggested.

Lines 475 to 477: Why is only the firing rate of pyramidal cells used to calculate BOLD? Mainly, the pyramidal cells' postsynaptic potential is reflected in M/EEG because of the number of pyramidal cells and their arrangement. However, that does not apply to BOLD. Here all neural masses contribute.

BOLD fluctuations are correlated with energy expenditure. The main energy expenditure comes from restoring the ion-gradients which sustain EPSPs. Because the sodium gradient is far from equilibrium, in comparison with the chloride gradient related to IPSPs, preserving the sodium gradient is thermodynamically expensive. In consequence, the energy expenditure related to the excitatory activity is higher in comparison to inhibitory activity (`http://doi.org/10.1016/j.neuroimage.2004.07.013`). In fact, there is some evidence that inhibitory activity does not produce a measurable BOLD response, because of the lower number of inhibitory synapses and their lower energy expenditure (`http://doi.org/10.1038/35023171`). In accordance with this, similar modeling works that calculate a BOLD signal from the activity of neural masses do it from the excitatory activity only (`http://doi.org/10.1016/j.cub.2018.07.083`).

We clarified this issue in the Methods section. In addition, we reproduced Figure 2 using a combined BOLD response. Three BOLD-like signals were generated, one signal for each neural mass and the signals were summed to produce a unique time series. We compared this approach with the original results (using only the firing rates of pyramidal neurons) and we found no noticeable differences. The results were appended as a Supplementary Figure.
