# Supplementary material for: Cholinergic neuromodulation of inhibitory interneurons facilitates functional integration in whole-brain models
Source: PLoS Comput Biol. 2021 Feb 18;17(2):e1008737. doi: 10.1371/journal.pcbi.1008737 (PMC7924765; doi:10.1371/journal.pcbi.1008737)
Supplement: Attachment — Submitted filename: ResponseTo Reviewers2.pdf [file pcbi.1008737.s010.pdf]

Reply to Reviewers: PCOMPBIOL-D-20-01747R1
Cholinergic neuromodulation of inhibitory interneurons facilitates functional integration in whole-brain models

Carlos Coronel-Oliveros, Rodrigo Cofré, Patricio Orio

January 21, 2021

We would like to thank the reviewers, once again, for the time spent in the critical reading of our revised work. They have really helped us to largely improve our manuscript. We are also happy to know that their concerns were satisfactorily addressed in our second version. Concerning the last minor issues raised by reviewer #2, we have prepared a new revised version of our manuscript, and the following is a point-by-point answer to the comments. We also took the opportunity to introduce minor corrections as well, properly identified in the highlighted version of the manuscript.

In the following, bold fonts are used for the referees' comments and regular fonts for our reply.

Reviewer 1

We are glad to know that all your concerns were addressed.

Reviewer 2

I found the introduction, in comparison to the overall length of the manuscript, a little bit lengthy. Making it more concise could improve the readability of the article.

Thanks for the suggestion. We have shortened the introduction by deleting some unnecessary remarks and clarifications that were repeated elsewhere.

l. 119: Which experimental findings are being referred to? Later in the manuscript the authors rather argue that they want to provide two time scales- I find this argument more convincing

We have added the relevant references at the end of the sentence, just to make the point clearer. Nevertheless, both arguments (the relationship to fMRI experiments and the study of two time scales) were equally important for us in this study.

Fig.1: It would make sense to the modification of the model more visible (e.g. using a color), to help the reader to directly see the changes

Fig. 3: It would be great to associate the red circles to the letters within the picture (e.g. B) to faster see which circle relates to which graph

Thanks again for the nice suggestions. Both were incorporated into the figures.

l.428- As this analysis is based on a simulation, I would rather not talk about "experiment"

We probably were not clear enough in our writing, but in the first half of the paragraph we were indeed referring to the *experimental* findings found in Refs. 33-36. On that basis, we built the hypothesis that was tested in this work through numerical simulations. After reading the paragraph again, we found that citing the references once again should be enough to avoid confusion.

l.598- It might be more advantageous for the authors' argument to name diseases with a known cholinergic deficit (e.g. Alzheimer's or Parkinson's disease dementia) and noradrenergic deficit (e.g. ADHD)

Again, a nice and useful suggestion that was incorporated into the text.

Discussion: I especially enjoyed reading the new version of the discussion

We are happy to know that, thank you!.
